# Chemotherapy with Alkylating Agents and Dental Anomalies in Children: A Systematic Review

**DOI:** 10.3390/jcm14031030

**Published:** 2025-02-06

**Authors:** Patrizia Gallenzi, Angela Malatesta, Edoardo Staderini, Federica Guglielmi

**Affiliations:** 1 Institute of Dental Clinic, A. Gemelli University Policlinic IRCCS, Catholic University of Sacred Heart, 00168 Rome, Italy; patrizia.gallenzi@unicatt.it (P.G.); edoardo.staderini@unicatt.it (E.S.); federica.guglielmi@unicatt.it (F.G.); 2 Postgraduate School of Orthodontics, Director: Prof. Massimo Cordaro, Catholic University of Sacred Heart, Largo A. Gemelli 8, 00168 Rome, Italy

**Keywords:** alkylating agents, tooth abnormalities, drug therapy

## Abstract

The aim of the systematic review is to analyze the type and the prevalence of dental side effects among cancer survivors treated with alkylating agents (AAs) during pediatric age. Moreover, the study aimed to investigate the association between the development of dental anomalies and the drug used or the tumor type. Four databases MEDLINE-PubMed, Web of Science, Scopus, and the Cochrane Central Register of Controlled Trials (CENTRAL) were searched from January 2024 to March 2024. All articles published up to March 2024 were evaluated. After removing duplicates, data extraction and risk of bias assessment using the Newcastle–Ottawa score were made. A summary of the overall strength of evidence available was performed using the “Grading of Recommendations Assessment, Development and Evaluation” (GRADE). Data were summarized using descriptive analysis as mean differences ± standard deviation or relative risks. Out of 2678 studies, the search identified five studies enrolled for the qualitative analysis of the data. Among 257 survivors, 155 (60.3%) reported: microdontia, agenesia, root shortening, enamel defects, and taurodontism. Microdontia occurred more frequently with other drugs compared to AAs. In conclusion, children treated with AAs showed microdontia (36.0%), root shortening (26.9%), and agenesis (23.5%). Secondly, the occurrence of dental anomalies was unaffected by drug treatment; thirdly, microdontia was the most frequent dental anomaly observed in both solid and lymphoproliferative tumors. This review was performed in accordance with the PRISMA guidelines. PROSPERO registration number CRD42023494560.

## 1. Introduction

### Rationale

Childhood cancers prevalence is increasing in the pediatric (0–14 years old) as well in adolescent (15–19 years old) populations [1]. As described by Crocetti et al. leukemias and lymphomas (16%) are the most frequent pediatric tumors [2], followed by malignant tumors of the Central Nervous System (CNS) (13%), tumors of the autonomic nervous system (8%), soft tissue sarcomas (7%), kidney tumors (5%), bone tumors (5%), retinoblastoma, thyroid tumors, and other rare forms of cancer [3,4]. Chemotherapy is one of the most used treatment approaches, although this type of drugs is associated with considerable side effects in growing patients, both in the short and long term [5]. Concerning dental-related events, an association between chemotherapy administration during childhood and Dental Developmental Abnormalities (DDA) in permanent dentition has been reported [6]. Alkylating agents (AAs) are a class of antineoplastic drugs which act by inhibiting the transcription of DNA into RNA, stopping the protein synthesis, and thereby inducing cells to undergo apoptosis.

AAs have been used for the treatment of leukemia and lymphomas [7] both in the adult and pediatric population [8]. However, the cytotoxic therapy in growing patients can affect the cells for tooth development, i.e., primitive mesenchymal cells and pulp pre-odontoblasts [2]. Dental side effects of AAs include enamel defects as well as other types of irreversible dental sequelae, such as tooth agenesis, root abnormalities, and alterations in tooth size and shape [2]. However, among the available antineoplastic therapies, a paucity of literature focused on the long-term dental effects of AAs.

Kaste et al. identified AAs as one of the primary causes of dental issues in childhood cancer survivors treated before the age of five; specifically, the cumulative dose of cyclophosphamide was associated with an increased risk of adverse dental outcomes [9,10]. Cyclophosphamide is an alkylating nitrogen mustard that acts an important role in polychemotherapy against a wide spectrum of tumors, both in adult and children [11], including leukemias, lymphomas [12,13], rhabdomyosarcoma [14], Ewing’s sarcoma [15], neuroblastoma [16], renal tumor, germ cell tumor, hepatic tumor, retinoblastoma, and brain tumors [17,18,19].

Current literature assessed the prevalence of any dental sequelae of chemotherapy matched with radiotherapy or other treatments [6]. However, there is a need for evidence discriminating the independent role of chemotherapy from other oncological treatments [20].

Therefore, the present systematic review aimed at assessing the role of chemotherapy with AAs in children in the development of dental anomalies.

## 2. Materials and Methods

### 2.1. Protocol and Registration

This systematic review was registered with the International Prospective Register of Systematic Reviews (PROSPERO) under registration number CRD42023494560 and follows the Preferred Reporting Items for Systematic Review and Meta-analyses guidelines (PRISMA) [21].

### 2.2. Search Strategy

The PECOS strategy used to guide the electronic search of the present review was:

Population (P) = cancer survivors treated with AAs (phase 3 studies); age: pediatric patients (0–17 years); type of cancers: all.

Exposure (E) = AAs submitted for antineoplastic treatment.

Comparison (C) = Dental abnormalities in patients receiving chemotherapy with AAs compared with other chemotherapy agents.

Outcomes (O) = (a) Type and prevalence of dental anomalies on permanent teeth, including microdontia, agenesis, enamel defects, taurodontism, short roots, root growth impairment, and delayed eruption associated with AAs; (b) association between the type of chemotherapy and dental anomalies; (c) frequencies of dental anomalies among different types of cancer.

Study design (S) = case–controls studies, cohort studies with prospective/retrospective design, or Randomized Clinical Trials (RCT).

According to the Population, Exposure, Comparison and Outcome (PECO) design format, the following clinical question was developed: “Can the exposure with AAs administered to pediatric patients induce dental abnormalities in the permanent dentition”?

The primary aim of this systematic review was to assess the type and the prevalence of dental side effects among long-term survivors undergoing chemotherapy with AAs.

The secondary aim was to estimate if the prevalence of dental abnormalities would change according to the type of chemotherapy drug used.

The tertiary aim was to compare the frequencies of dental anomalies according to tumor type.

### 2.3. Eligibility Criteria

The following inclusion criteria were employed for this systematic review: (1) studies with pediatric cancer survivors aged between 0 and 17 years of both genders, with no year or language restriction, and published until March 2024; (2) scientific studies with the clear purpose to assess the relationship between dental anomalies and AAs.

The following were the exclusion criteria: (1) studies that were not research articles, such as reviews, meta-analysis, book chapters, personal opinions, and case reports, and (2) studies on adult cancer survivors [Table 1].

### 2.4. Information Sources and Search

The literature search was conducted from January 2024 to March 2024. All articles published up to March 2024 were evaluated. The following electronic databases were systematically searched: MEDLINE-PubMed, Web of Science, Scopus, and the Cochrane Central Register of Controlled Trials (CENTRAL). References for included articles were subsequently screened as well.

### 2.5. Data Collection Process

Data were first extracted independently and duplicated by two reviewers using designed data extraction forms (EndNote^®^ X9, Clarivate, Philadelphia, PA, USA). Collected studies were imported into EndNote^®^ for deduplication by the same reviewers. Microsoft Excel^®^ v.2021 was used to screen titles and abstracts and then to scrutinize the data extracted from the full text articles.

### 2.6. Study Selection

After the deduplication performed on EndNote^®^ X9, (Clarivate) titles and abstracts’ screening was conducted independently and in duplicate by two independent and expert reviewers. Then, the same reviewers performed the assessment of the full texts of the included articles. Any disagreements between authors were solved through discussion with a third investigator. Agreement between reviewers was evaluated using Cohen’s Kappa.

### 2.7. Data Items

The following variables which were extracted from each selected article included: study type, year of publication, sample size, population details (including age), type of cancer diagnosis, type and details of antineoplastic treatment, chemotherapy drugs used (AAs), and prevalence of dental anomalies among survivors.

### 2.8. Outcomes

Primary, secondary, and tertiary outcomes of this review were either binary or continuous and were expressed as Relative Risks (RR) or Mean Differences (MD), respectively, with their corresponding 95% Confidence Intervals (CI). Data were summarized using descriptive analysis as MD and standard deviation, or median and interquartile range.

### 2.9. Risk of Bias in Individual Studies and Quality of Evidence

To assess the risk of bias of all the included articles, RoB-2 Cochrane risk-of-bias tool for RCT and the Newcastle–Ottawa Scale (NOS) for non-RCT were used [22].

According to the Newcastle–Ottawa scale, the risk of bias was scored as high, moderate, or low for each study, with the last category indicating either lack of information or uncertainty over the potential for bias [23].

A summary of the overall strength of evidence available was performed using “Grading of Recommendations Assessment, Development and Evaluation” (GRADE) and summarized with Cochrane’s Review Manager Software (RevMan 5.4.1) [24].

## 3. Results

### 3.1. Study Selection

The present review was restricted to studies that investigated dental late sequalae among survivors subjected to AAs. The initial electronic search resulted in a total of 2678 titles and 22 studies were found by manual search. After the elimination of duplicate articles, a total of 2673 titles were screened for possible inclusion. A total of 2599 articles were removed based on their title and abstract; therefore, 74 full-text articles were assessed. After full-text evaluation, 69 articles were excluded. Among these, 20 were excluded because their design did not fit the inclusion criteria; 49 were excluded because it was not possible to determine the specific type of treatment the patients underwent, including whether they received chemotherapy, radiotherapy, or a combination of both. Therefore, five were included in the review. After screening references of the selected articles, no additional publications were recovered.

**Figure 1:**
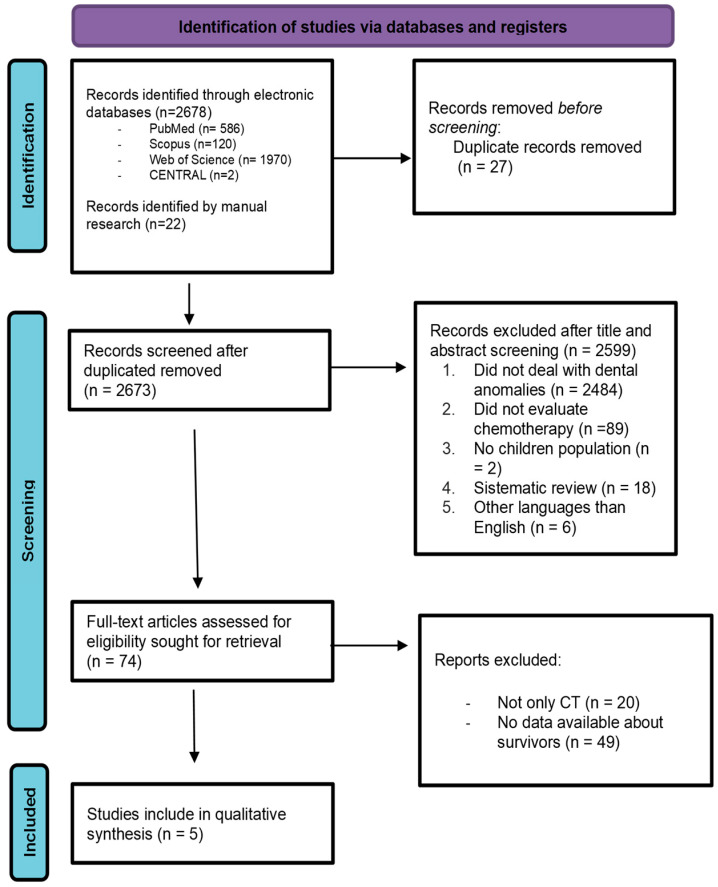
PRISMA flowchart that includes identification of studies, screening, eligibility, and inclusion data.

In conclusion, only five studies were identified as eligible for inclusion in this review. [Figure 1] Cohen’s Kappa value of 0.81 was obtained with perfect agreement among reviewers.

### 3.2. Quality Individual Assessment

At the end, all the included studies were non RCT; therefore, the eNOS were used [Table 2]. All studies analyzed have been assessed as having a moderate risk of bias, indicating that they are generally well-conducted, but there were some limitations in their study design. The limitations of the included studies are mainly related to the absence of a control group of unexposed patients, which is common in cohort studies.

### 3.3. Included Studies Characteristics

Overall, the included studies were very heterogeneous since the same outcomes were reported using different parameters. The included articles reported the number of patients undergoing chemotherapy and, when possible, the number of patients undergoing AAs treatment. The descriptive analysis of the five included studies is summarized in Table 3.

#### 3.3.1. Characteristics of Participants

The participants of these studies were treated for hematological malignances, CNS solid tumors (ST) and non-CNS ST, B-cell germinal tumor, Wilms tumor, hepatoblastoma, neuroblastoma, primitive neuroectodermal tumor, rhabdomyosarcoma, medulloblastoma, anaplastic ependymoma, and retinoblastoma at a young age (0–17 years). During growth several dental abnormalities appeared on their permanent teeth. The age of patients at the beginning of treatment was between 0 and 17 years old, while the age at the follow-up was heterogeneous as reported in Table 3.

#### 3.3.2. Characteristics of Interventions

Among participants in the included studies, 325 received chemotherapy with AAs such as cyclophosphamide, cisplatin, carboplatin, busulfan, melphalan, treosulfan, dacarbazine, temozolomide, thiotepa, isosfamide, or mitomicyn-C.

#### 3.3.3. Type and Prevalence of Dental Anomalies Related to AAs

**Figure 2:**
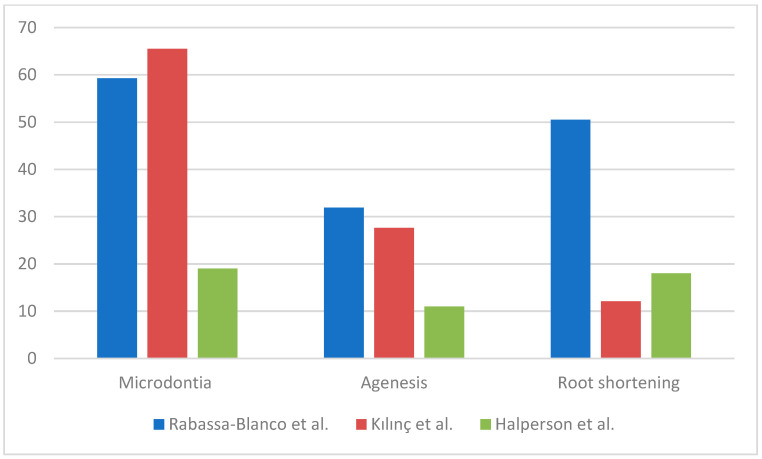
Prevalence and type of dental anomalies.

Among the included articles, Stolze et al. did not report the crude prevalence of dental abnormalities among those who had undergone AAs, so the rate of anomalies was calculated out of 257 people [3]. We found that 155 survivors submitted to AAs out of 257 (60.3%) reported the following dental abnormalities: microdontia, agenesia, root shortening/malformation, enamel defects, taurodontism. The authors reported a dose-dependent risk of dental anomalies related to alkylating exposure with RRs of 1.46 (95% CI, 0.78 to 2.73), 1.89 (95% CI, 1.03 to 3.47), and 2.61 (95% CI, 1.39 to 4.91), for >0–3999 mg/m^2^, 4000–9999 mg/m^2^, and ≥10,000 mg/m^2^, respectively (Ptrend = 0.390) [3]. Jodłowska et al. affirmed that the higher the administered dose of alkylating agents, the higher the risk of developing dental disorders [20]. Rabassa-Blanco et al. concluded that patients who took cyclophosphamide, cisplatin, or carboplatin present an RR of developing moderate lesion of 3.36 and an RR of 2.29 of presenting severe ones. They found that the most prevalent abnormalities were microdontia (59.3%), root shortening (50.5%), and agenesis (31.9%) (Figure 2) [2]. Teeth affected by microdontia and short roots were mostly first and second premolars, followed by permanent second molars and lateral incisors [2,3,17]. Halperson et al. found that 36 (43%) patients out of the 83 undergoing chemotherapy reported the following dental abnormalities: microdontia (19%); root changes (18%); and hypoplasia (13%) [25].

#### 3.3.4. Age at Chemotherapy Administration

The study conducted by Rabassa Blanco et al. involved patients treated with chemotherapy at the age of 0–5 years [2]; similarly, the participants in Kılınç’s study also ranged in age from 0.7 to 9 years [17]. Patients in the studies of Stolze et al. and Halperson et al. have undergone chemotherapy before the age of 17.3 [25]. Except for microdontia, long duration of treatment was not associated with a higher number of dental lesions [2]. Jodlowska et al. enrolled patients starting chemotherapy before the age of 10. They observed that survivors starting their treatment between four and seven years old did not present severe dental anomalies; possibly because they received the anticancer treatment in a period which is not considered critical for dental tissues developmental, even though the therapy could last long [20]. All authors agree that survivors who received chemotherapy before 5 years of age had a statistically increased risk of developing a dental developmental disorder in comparison to survivors older than 5 years of age at diagnosis [3]. There is a relative risk of 2.19 for patients who start therapy before 36 months [2].

#### 3.3.5. Correlation Between Dental Anomalies and Drug Therapy

**Figure 3:**
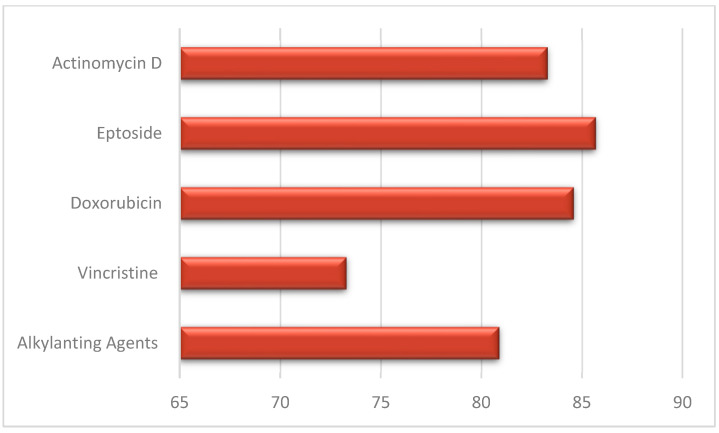
Dental anomalies and drug therapy.

Jodłowska et al. performed an analysis of the effects on dentition separately for different drugs: patients treated with AAs showed a prevalence of dental anomalies of 80.9% ± 3.4 while those treated with vincristine 73.3% and those treated with doxorubicin/actinomycin D/eptoside 84.5% ± 1.2 (Figure 3) [20]. Rabassa-Blanco et al. showed that plant alkaloids and topoisomerase inhibitors cause root shortening (RS) with a similar prevalence than AAs (respectively, 49.5–59.5–50.5%), and that cytotoxic antibiotics cause agenesis more often than AAs (35.5–31.9%). A higher occurrence of microdontia was reported with other drug use instead of AAs (65.8–59.3%) [2]. According to Halperson et al., no significant differences were observed between the number/type of dental malformations and the chemotherapeutic drugs used [25].

#### 3.3.6. Relation of Dental Anomalies Among Different Types of Cancer

**Figure 4:**
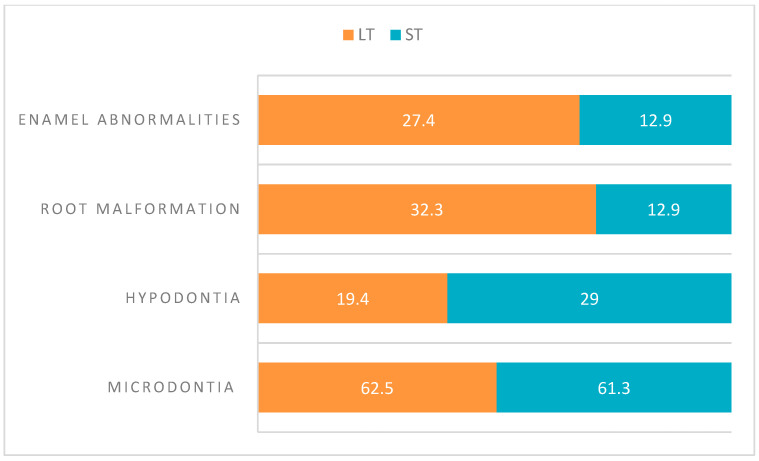
Dental anomalies among type of cancer. (LT: lymphoproliferative tumors; ST: solid tumors).

Kilinç describes that more than 80% of patients with LT and ST presented dental abnormalities: microdontia showed an overlapping prevalence in both groups (62.5% LT; 61.3% ST), while hypodontia (19.4% LT, 29% ST), root malformations (32.3% LT; 12.9% ST), and enamel abnormalities (27.4% LT; 12.9% ST) had a different prevalence between LT and ST [17] [Figure 4].

### 3.4. GRADE Assessment

The quality of evidence was assessed as low for the second and third outcomes; the main limitation is represented by the study design of eligible studies. Concerning the first outcome, which focused on the type and prevalence of dental side effects, the quality of evidence was upgraded from low to moderate [Table 4].

This upgrade was justified by two reasons related to the dose–response relationship and the significant size effect:

Stolze et al. reported a dose-dependent risk of dental anomalies related to alkylating exposure with RR of 2.61 (95% CI, 1.39 to 4.91).

Rabassa-Blanco et al. concluded that patients who took cyclophosphamide, cisplatin, or carboplatin present a RR of developing moderate lesion of 3.36 and a RR of 2.29 of presenting severe ones.

## 4. Discussion

The results of this systematic review showed that patients treated with AAs during childhood have an increased risk of developing dental abnormalities in permanent dentition, with an overall RR of 3.36 [2,3]. It is well-known that chemotherapeutic drugs act on cells with a high replication index, destroying, through various mechanisms of action, tumor cells as well as healthy cells of the body, indifferently [26]. For this reason, it can be hypothesized that dental development could be compromised in various ways depending on the child’s age at the treatment, the type and dosage of treatment protocol, and the prescribed drugs [27]. The most prevalent dental anomalies are microdontia, root shortening, and hypodontia/agenesis. The included studies are consistent in indicating that the younger the patient the higher the severity and type of dental abnormalities; in fact, according to the tooth development, some patients may present with twice the risk compared to those who started therapy later [2,3,20,25]. When children start anticancer therapy by the age of 42 months, microdontic teeth are observed, while anomalies such as enamel defect and root malformation are more common among patients treated after the age of 5 [20,28]. Similarly, hypodontia is more often found among survivors who took chemotherapy before 5 years old [20], even if Proc et al. did not find a statistically significant association between age at the beginning of therapy and the number of missing teeth [28]. The study conducted by Hsieh et al. reported that participants who had received 7500 mg/m^2^ or more cyclophosphamide showed an increase of about 13 points in Holtta’s Defect Index (HDI) compared with those who had not been treated with this drug (*p* = 0.01). The HDI measures dental abnormalities such as microdontia, tooth aplasia, and root/crown ratio [10]. The absence of this association may be attributed to the strong genetic component involved in the etiology of dental agenesis [29]. Enamel hypomineralization/hypoplasia have been hypothesized to be attributed to AA since they alter the function of ameloblast and the calcium transports through microtubules [2]. The most frequently affected teeth by hypodontia are second premolars [18]. The calcification of the first premolars, the second premolars and second molars begin between the ages of 1.5–2, 2–2.5, and 2.5–3 years, respectively [2]. Pedersen et al. also identified an association between microdontia and exposure to chemotherapy. Similarly to hypomineralization, the occurrence of microdontia was related to the stage of dental development at which chemotherapy was started [27].

Although there is agreement in the literature on the correlation between AAs and dental abnormalities, the present systematic review showed that these drugs are not the only ones associated with dental side effects. The data analyzed from the studies included in the present review indicate that other drugs submitted to patients in the chemotherapy protocol are involved in the occurrence of dental abnormalities, with a similar or higher rate [2,27]. Halperson et al. reported that the type and the number of DDA did not differ significantly according to chemotherapy agent; however, it is also difficult to attribute specific influence on dental development to any single drugs because of the combination of multi-agent chemotherapy [25]. As shown by Kılınç et al., AAs are usually part of a polychemotherapy protocol, so it is difficult to assess an association between chemotherapy agents and DDA [17]. Moreover, ministerial guidelines for the promotion of oral health and the prevention of oral diseases in Italy suggested to inform parents and caregivers of patients undergoing AA (particularly cyclophosphamide) about the side effects of AAs on dental maturity.

### 4.1. Strengths

The present study has some strengths, in fact to the best of our knowledge, the present study is the first systematic review focusing on the side effects of AAs on dental development. Its protocol has registered a priori in PROSPERO and the methodology has been strictly followed.

### 4.2. Limitations

There are also some limitations: firstly, this systematic review included only non-RCT studies as no RCT study on the topic was retrieved; however, it should be noted that all included studies appeared to have a moderate risk of bias. In addition, the limited number of studies included does not allow for robust assessments of heterogeneity. Moreover, the quality of evidence is low, mainly because these drugs are included within multiagent chemotherapy protocols to create synergism and additive effects, making it difficult to assess the effect of specific drugs alone.

### 4.3. External Validity

The data obtained from this study indicates that there is an association between dental abnormalities and AAs; therefore, it suggests patients undergo a pediatric dental examination before the start of chemotherapy and subsequent periodic check-up appointments to intercept the occurrence of dental abnormalities and set up the appropriate management.

## 5. Conclusions

Long-term survivors undergoing AAs therapy may report dental abnormalities as a late effect of chemotherapy, with a prevalence between 18 and 65%. Microdontia (36.0%), root shortening (26.9%), and agenesis (23.5%) appeared to be the most frequent dental anomalies.

The prevalence of dental anomalies does not change depending on the drugs used. In addition, it is difficult to determine the independent effects of each antineoplastic agent and AAs on dental anomalies, or to discriminate between the effects of different treatment strategies, because most childhood cancers are treated with a combination of multiple chemotherapeutic drugs to create synergistic and additive effects. Further studies are encouraged to better evaluate the effect of AAs on dental development, regardless of concomitant administration of other drugs or antineoplastic treatments.

Few data on the distribution of AAs-treated dental abnormalities and tumor type are available; however, it is possible to show an overlapping prevalence of microdontia in ST and LT while root malformations and enamel anomalies appear more frequent in LTs and hypodontia in STs.

## Figures and Tables

**Table 1:** Inclusion and exclusion criteria.

Inclusion Criteria	Exclusion Criteria
Pediatric cancer survivors aged between 0 and 17 year of both genders	Not research articles
No year or language restriction	Studies on adult cancer survivors.
Studies published until March 2024	
Studies with the purpose of assessing the relationship between dental anomalies and AAs.	

**Table 2:** Newcastle–Ottawa Scale score.

Study	Selection				Comparability	Outcomes			Total	Risk of Bias
	Representativeness of Exposed Cohort	Selection of Non-Exposed Cohort	Ascertainment of Exposure	Outcome Not Present at the Start of the Study	Comparability of Cohorts on the Basis of the Design or Analysis Controlled for Confounders	Assessment of Outcome	Length of Follow-Up	Adequacy of Follow-Up		
Rabassa-Blanco et al. [2]	*	-	*	-	*	*	*	*	6/9	Moderate
Kılınç et al. [17]	*	-	*	*	*	*	*	*	7/9	Moderate
Halperson et al. [25]	*	-	*	-	*	*	*	*	6/9	Moderate
Jodłowska et al. [20]	*	-	*	-	*	*	*	*	6/9	Moderate
Stolze et al. [3]	*	-	*	-	*	*	*	*	6/9	Moderate

*: yes; -: no.

**Table 3:** Characteristics of included studies.

Author	Publication (Year)	Study Design	Diagnosis	Age (Beginning of CT)	Drug Therapy	Treatment Duration	Discrimination Between Those Who Have Been Treated with RT and CT?	AAs Only	Patient Treated with AAs	Age at the Follow-Up	Dental Anomalies/Patients Treated with AAs	Type of Dental Anomalies
Rabassa-Blanco et al. [2], *Oral Dis*	2022	Single-center retrospective cohort study	Leukemias and lymphomas 45; Solid tumors excluding CNS 22; Solid tumors CNS 42	0–5 y.o.	CT, CT + RT, HSCT	1.9 y	Yes	No	91	12–18 y.o.	51/91	Microdontia 59%; Agenesis (31.9%); Root shortening (50.5%)
Kılınç et al. [17], *Turk J Haematol*	2019	Case–Control study	Leukemia, Lymphoma, and Langerhans cell histiocytosis, Solid tumors	0.9–7 y.o.	CT, CT + RT	N.A.	Yes	No	57	>8 y.o.	47/57	Microdontia (65.5%); Hypodontia (27.6%); Root malformation (12.1%)
Halperson et al. [25], *Sci Rep*	2022	Cross-sectional study	Acute lymphocytic leukemia, Acute myelocytic leukemia, Non-Hodgkin Lymphoma, Hodgkin Lymphoma, Sarcoma, Neuroblastoma, Other solid tumors, Hematological condition	0.1–17–7 y.o.	CT, CT + RT	N.A.	Yes	No	83	0–18 y.o.	36/83	Microdontia (19%); Root changes (18%); Hypoplasia (13%)
Jodłowska et al. [20]; *Int J Environ Res Public Health*	2022	Cross-sectional study	Nephroblastoma, Neuroblastoma, Medulloblastoma, Hepatoblastoma, Infantile fibrosarcoma, sarcoma, teratoma malignum, embryonal primitive neuroectodermal tumor (PNET)/Ewing sarcoma (ES), yolk sac tumor, clear cell sarcoma, astrocytoma pilocyticum; Hematological cancers, acute lymphoblastic leukemia, Hodgkin lymphoma, myelomonocytic lymphoma.	<10 y.o.	CT, RT	36.57 months (CP = min 11/max 91; CBDCA = min 4/max 88)	Yes	No	26	6–17 y.o.	21/26 (10 treated with CP; 11 treated with CBDCA)	Agenesis; Microdontia; Crown reduction size; Tauroodontism
Stolze et al. [3]; *Cancers*	2021	Cross-sectional study	Hematological malignancies	0–17 y.o.	CT, CT + RT	N.A.	Yes	No	68	30.3 y.o. (min 16.8–max 51.6 y.o)	N.A.	Agenesis; Microdontia (% N.A.)

Abbreviations: CT: Chemotherapy. RT: Radiotherapy. CNS: Central Nervous System. AAs: Alkylating Agents. HSCT: Hematopoietic Stem Cell Transplantation. N.A.: Not Assigned. Y.O: Years Old. CP: Cyclophosphamide. CBDCA: Carboplatin.

**Table 4:** GRADE summary.

N° of Studies	Study Design	Limitations	Inconsistency	Indirectness	Imprecision	Other Consideration	Quality of Evidence
Type and prevalence of dental side effects among long-term survivors undergoing chemotherapy with AAs							
5	Cross-sectional (3 studies); Case–control (1 study); Retrospective cohort (1 study)	No serious limitations	No serious inconsistency	No serious indirectness	No serious imprecision	Upgrade from low(non-RCT design) to moderate quality of evidence for the presence of a dose–response gradient and for a large magnitude of effect (RR > 2)	⊕⊕⊕O Moderate
Correlation between dental anomalies and drug therapy							
5	Cross-sectional (3 studies); Case–control (1 study); Retrospective cohort (1 study)	No serious limitations	No serious inconsistency	No serious indirectness	No serious imprecision	None	⊕⊕OO Low
Relation of dental anomalies among different types of cancer							
5	Cross-sectional (3 studies); Case–control (1 study); Retrospective cohort (1 study)	No serious limitations	No serious inconsistency	No serious indirectness	No serious imprecision	None	⊕⊕OO Low
